# Prevalence and intensity of soil transmitted helminths among school children of Mendera Elementary School, Jimma, Southwest Ethiopia

**DOI:** 10.11604/pamj.2017.27.88.8817

**Published:** 2017-06-06

**Authors:** Ephrem Tefera, Tariku Belay, Seleshi Kebede Mekonnen, Ahmed Zeynudin, Tefera Belachew

**Affiliations:** 1 Department of Medical Laboratory Sciences, College of Health and Medical Sciences, Haramaya University, Harar, Ethiopia; 2 Department of Medical Laboratory Sciences, College of Public Health and Medical Sciences, Jimma University, P.O. Box 378, Jimma, Ethiopia; 3 Department of Population and Family Health, College of Public Health and Medical Sciences, Jimma University, P.O. Box 378, Jimma, Ethiopia

**Keywords:** Prevalence, intensity, soil transmitted helminths, school children, Ethiopia

## Abstract

**Introduction:**

Soil transmitted helminths are wide spread in developing countries and in Ethiopia the prevalence of STHs varies in different parts of the country. The aim of this study was to determine the prevalence and intensity of soil transmitted helminths among school children of Mendera Elementary School Jimma town, Southwestern Ethiopia.

**Methods:**

A cross-sectional study was conducted between March 29 and April 9, 2010 to determine the prevalence and intensity of soil transmitted helminths among elementary school children. The study participants were randomly selected from class enrollment list after proportional allocation of the total sample size to each grade. Data about the background characteristics were collected using structured questionnaire. The stool samples were examined by McMaster method for the egg count which was used to determine intensity of infection. Data were analyzed using SPSS for windows version 16 and p-value less than 5% was considered as statistically significant.

**Results:**

Of the total 715 stool specimens examined, 346 were positive for at least one intestinal parasite making the prevalence 48.4%. The most prevalent parasites were Ascaris *lumbricoides* 169 (23.6%) and *Trichuris trichiura* 165 (23.1%). The prevalence of soil transmitted helminth in this study was 45.6% (326/715). There was statistically significant difference in the prevalence of Trichuriasis between those who use latrine always and who use sometimes (p = 0.010). Females are two times more likely to be positive for Ascaris than males (p = 0.039). Majority of the students had light infection of soil transmitted helminths and none of them had heavy intensity of infection of Trichuriasis and hookworms.

**Conclusion:**

Nearly half of the school children were infected with at least one STHs and majority of the students had light infection of soil transmitted helminths. Students who did not wash their hands after defecation were three times more likely to be positive for Ascaris infection than those who washed their hands after defecation. Therefore, measures like health information dissemination on the advantage of washing hands after defecation and on proper use of latrine should be taken into account to alleviate the problem.

## Introduction

Intestinal helminthic infections are important public health problems in tropical countries. Unlike in developed countries where efficient control, urbanization and other socioeconomic factors have created better conditions for the decline in prevalence of intestinal parasitic infections, these infections still continue to be a major health problem in the third world countries particularly soil transmitted helminthic infections have been recognized as important public health problems in many developing countries [1]. The main parasites that cause soil transmitted helminthiases *Ascaris lumbricoides*, *Trichuris trichiura*, and the hookworms *(Ancylostoma duodenale* and *Necator americanus)* are the most widespread species from the soil transmitted helminths. An estimated 4.5 billion individuals are at risk of STHs and as many as 1.4 billion individuals might be infected with *A. Lumbricoides*, close to 1.05 billion with *T. Trichiura*, and more than 1.3 billion with hookworms [2, 3]. The greatest numbers of soil-transmitted helminths infections occur in tropical and subtropical regions of Asia, especially China, India and Southeast Asia, as well as sub-Saharan Africa. Of the 1-2 billion soil-transmitted helminths infections worldwide, approximately 300 million infections result in severe morbidity, which are associated with the heaviest worm burdens [4].

In Ethiopia parasitic helminthic infections are the second most predominant causes of outpatient morbidity [5]. Several studies indicated that the prevalence of parasitic infections were high in the lower altitudes including southwestern Ethiopia [6]. Ethiopia has one of the lowest quality drinking water supply and latrine coverage in the world. Many reports illustrated that *A. Lumbricoides* is the most prevalent intestinal parasite in different communities usually occurring together with Trichuris infections [7]. Hookworm infection, is also public health problem though the magnitude is lesser compared to Ascariasis [6–8]. Infection intensity is a key factor in understanding the morbidity of STH; although light infections are often asymptomatic, heavy infections cause an array of morbidities, including dietary deficiencies and delayed physical and cognitive development [9]. Additionally, Hookworm and *T. Trichiura* infections contribute to iron-deficiency anemia [9]. Moreover, estimates of the global burden due to STH range between 4.5 million and 39 million disability-adjusted life-years [9]. Several studies depicting the prevalence of intestinal parasites in general and the prevalence of soil transmitted helminths in particular were carried out in Ethiopia [10–12] and in different countries in the world [13–16] with varying degree of prevalence and intensity of infections. In a recent study [17] which was conducted in Jimma town to determine the prevalence of intestinal parasites 83% had at least one intestinal parasitic infection. In the same study the prevalence of *T. trichiura, A. lumbricoides,* and hookworms were 60.9, 40.9 and 17.5% respectively. The prevalence of *T. Trichiura* was highest among school age children (73.5%) followed by *A. Lumbricoides* (52.1%) and Hookworms (25.6%). Overall the prevalence of soil transmitted helminth in school age children was 58%. Even though, prevalence studies have been carried out in the study area no intensity study is conducted. So the purpose of the current study was to determine the prevalence and intensity of soil transmitted helminths among students of Mendera Elementary School.

## Methods

This cross-sectional study was conducted between March 29 and April 9, 2010 in Mendera Elementary school located in Jimma town. The town is located at 335Km away from Addis Ababa to the southwestern Ethiopia. The total area of the town is 102 Km^2^. The town is found in Jimma zone, one of the eleven zones in Oromia Regional State [18]. According to the 2007, population and housing census the town has a total population of 120,600, of which 60,590 are males and 60,010 are females. Temperature ranges from 12-30°C with a mean daily temperature of 19°C and the average annual rainfall is 800-2500mm. The town has an altitude of 1720-2010 m above sea level. The latrine coverage of the town is 91% it has water coverage of 84% [1,19].

Seven hundred fifteen students were selected from the school to participate in the study. The sample size was determined with the assumption that the proportion of STHs among the school children is 58% which is obtained from a study conducted by Amare *et al.* in 2007 [17]. By considering a 95% confidence level and a 5% expected margin of error the required sample size was 374. To minimize errors arising from the probable occurrence of non compliance, 10% of the sample size was added to the calculated sample size thereby making it to be 411. Since Mendera Elementary School was selected using cluster sampling technique from 12 governmental elementary schools found in the town we assumed a design effect of 2 that made the actual sample size 822. Out of the 822 sampled students 715 were voluntary to be involved in the study thereby making the response rate to be 87%.

The total sample size were allocated to different grades i.e. grade 1 to 8 of Mendera Elementary School proportional to size of each grade and the sampling frame was the students' enrollment list. Then the study subjects were selected from the list at random using random number table.

Two trained data collectors collected all the necessary background data by using structured questionnaire. After giving adequate instruction, each study participant was provided with a stool cup, applicator stick and soft tissue paper (for cleaning) to bring 3gm of fresh stool sample of their own, which was sufficient for direct wet mount as well as for the McMaster method to count the eggs. Finally each sample was labeled and transported to side lab belonging to the Department of Medical Laboratory Sciences and Pathology, Jimma University within half an hour together with filled questionnaire for processing and examination. All the 715 stool samples collected were examined by the McMaster method by two experienced Laboratory Technologists to look for STHs eggs as well as to count. Data collection and microscopic examinations were supervised regularly. According to WHO guidelines, intensity of infection was classified as ''light'', ''moderate'' or ''heavy'' on the basis of fecal egg count [20] (Table 1).

**Table 1:** Egg counts (egg per gram of feces) used to describe intensity of infection

Causative Pathogen	Intensity of infection (egg count per gram)		
	Light	Moderate	Heavy
*A. lumbricoides*	1 - 4999	5000 – 9999	≥ 10 000
*T. trichiura*	1 - 999	1000 - 9999	≥ 10 000
Hookworm	1 - 1999	2000 - 3999	≥ 4 000

Data were entered into a computer then cleaned and analyzed using SPSS windows version 16. Descriptive statistical methods were used to summarize the collected data. The intensity of infection was determined for *A. lumbricoides, T. trichiura* and Hookworms and expressed as egg per gram (epg) of feces for each student. The associations between dependent categorical variable and independent categorical variables were assessed using Chi-square. Logistic regression was used to evaluate the effect of factors on the probability of soil transmitted helminthic infection. P-value <0.05 was taken as indicator of significant association.

Ethical clearance was obtained from Ethical Review Board of Jimma University. Before the study was started, parents or guardians of the study subjects were gathered in different times and they were clarified about the objective of the study and finally signed on the consent form. In addition, verbal ascent was obtained from each of the study participants before they gave stool sample. All students positive for intestinal parasites were treated using appropriate drugs.

## Results

**Figure 1:**
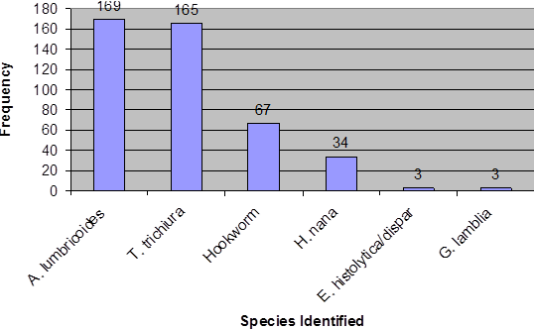
Number of students infected with intestinal parasites

Out of 822 randomly selected students 715 (87%) were volunteer to participate in the study and provide stool sample. Majority of the study participants were females (60.6%). Larger proportions (59.9%) were in the age range of 10-14 years, 48.4% were first cycle students (grade 1 to 4) and 51.6% were in grades 5 to 8 (Second cycle) (Table 2). Of the 715 stool specimens examined, 346 were positive for at least one intestinal parasite resulting in prevalence of 48.4%. The most prevalent parasites were *Ascaris lumbricoides* 169 (23.6%) and *Trichuris trichiura* 165 (23.1%). The prevalence of soil transmitted helminth in this study was 45.6% (326/715) (Figure 1). Of the 326 soil transmitted helminths infected students, 256 students were infected with either of the three STHs (Al, Tt, Hw), 65 students were infected with two of the three STHs (Al, Tt, Hw) and only 5 students were infected with all of the three STHs (Al, Tt, Hw). Hence the prevalence of single, double and triple infection by soil transmitted helminths were 35.8%, 9.1% and 0.7% respectively.

**Table 2:** Socio-demographic characteristics of students of Mendera Elementary School, Jimma, 2010

Variables		Frequency	Percentage
**Sex**	Male	282	39.4
	Female	433	60.6
**Age group**	5-9	165	23.1
	10-14	428	59.9
	15-19	121	16.9
	20^+^	1	0.1
**Ethnicity**	Oromo	369	51.6
	Amhara	130	18.2
	Dawro	52	7.3
	Yem	47	6.5
	Keffa	43	6.0
	Gurage	34	4.7
	Tigre	20	2.8
	Other	20	2.8
**Religion**	Orthodox	359	50.2
	Protestant	144	20.1
	Catholic	5	0.7
	Muslim	207	29.0

The prevalence of *Ascaris lumbricoides* was 23.6% (169/715) and there was no significant gender difference (p = 0.08) as well as between different age categories (p = 0.32). Similarly, the prevalence of *Trichuris trichiura* among the school children was 23.1% (165/715) and it was not significantly associated with gender and age of study participants. The prevalence of hookworms was 9.4% (67/715) (Table 3). Prevalence of the two soil transmitted helminths (*A. Lumbricoides* and *T. Trichiura* ) were not significantly associated with predisposing factors such as finger nail status, hand washing habit before meal, hand washing habit after defecation, presence or absence of latrine, the habit of eating uncooked vegetables and water source for drinking (p > 0.05).

**Table 3:** Frequency of soil transmitted helminths in relation to sex among students of Mendera Elementary School, Jimma, 2010

Variables		Result of Stool Examination		P-value
		Number (%) negative	Number (%) positive	
***A. lumbricoides***				
**Sex**	**Male**	225(79.8%)	57(20.2%)	0.08
	**Female**	321(74.1%)	112(25.9%)	
	**Total**	546(76.4%)	169(23.6%)	
***T. trichiura***				
**Sex**	**Male**	208(73.8%)	74(26.2%)	0.11
	**Female**	342(79.0%)	91(21.0%)	
	**Total**	550(76.9%)	165(23.1%)	
**Hookworms**				
**Sex**	**Male**	258(91.5%)	24(8.5%)	0.52
	**Female**	390(90.1%)	43(9.9%)	
	**Total**	648(90.6%)	67(9.4%)	

**Figure 2:**
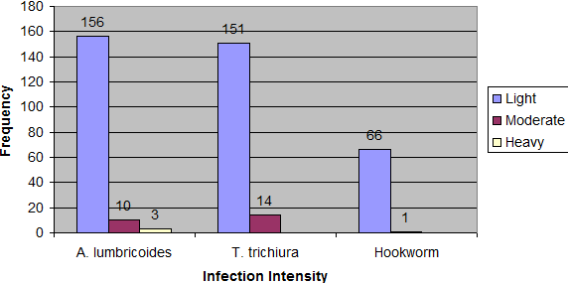
Intensity of soil transmitted helminths infection

Of the 707 students who have latrine in their dwelling compound, 673 use latrine always and 34 students use latrine sometimes. Of these 707 students 163 were infected with *T. trichiura* and the prevalence was significantly different between those who use latrine always and who use sometimes (p = 0.01). Hookworm infection was not significantly associated with shoe wearing habit and water source for drinking. Similarly, gender, finger nail status, the habit of eating uncooked vegetables, presence or absence of latrine, latrine usage, hand washing before meal, hand washing after defecation and water source for drinking were not significantly associated with the prevalence *H. Nana* (p > 0.05). However, statistically significant difference of *H. nana* infection were observed among the different age groups (p = 0.001). Iintensity of ascariasis, trichuriasis, and hookworm infections summarized in Figure 2 shows that there were no heavy infections of Trichuriasis and Hookworm.

Of the 169 *A. Lumbricoides* infected students, 156 had light infection, 10 had moderate infection and only 3 had heavy infection. Of the 165 *T. Trichiura* infected students 151 had light infection and 14 had moderate infection. Similarly, of the 67 Hookworm infected students 66 had light infection and only 1 student had moderate infection. The arithmetic mean (+ SD) egg count for each species of soil transmitted helminth is indicated as follows: *A. Lumbricoides* 1994.08 (±4305.488), *T. Trichiura* 429.09 (±658.900) and Hookworms 388.06 (±382.82).

Summary result in Table 4 shows that females are 2 times more likely to be positive for Ascaris than males (adjusted OR 1.69 and 95% C.I. is 1.03 - 2.78) and (p = 0.039). Similarly not washing hands after defecation increases the odds of ascaris infection 3 times compared to the habit of hand washing after defecation (adjusted OR = 2.92 and 95% CI is 1.09- 7.83). Students of higher grade are 25% less likely to develop hookworm infection than students of lower grade (adjusted OR 0.75 and 95% C.I. is 0.62-0.90) (Table 5).

**Table 4:** Parameter estimates from multivariable logistic regression mode predicting the probability of Ascaris infection among students of Mendera Elementary School, Jimma, 2010

Predictors of Ascaris infection	B	P value	Adjusted OR	95.0% C.I	
				Lower	Upper
Male			1.00		
Sex	0.53	0.039*	1.69	1.03	2.78
Age(years)	-0.11	0.123	0.90	0.79	1.03
Grades of the students	0.15	0.057	1.16	1.00	1.36
Finger nail status (trimmed)			1.00		
Finger nail status	-0.07	0.766	0.93	0.58	1.49
Latrine usage (Always)			1.00		
Latrine usage	-0.18	0.727	0.83	0.30	2.31
Hand washing after defecation (Yes)			1.00		
Hand washing after defecation	1.07	0.033*	2.92	1.09	7.83
Eating uncooked vegetables (No)			1.00		
Eating uncooked vegetables	-0.39	0.129	0.68	0.41	1.12
Water for drinking (Tap water)			1.00		
Other* source for drinking	0.13	0.809	1.14	0.41	3.19

Other *like Birrie, spring, combination of Tap and Birrie, Tap and spring

**Table 5:** Parameter estimates from multivariable logistic regression model predicting the probability of Hookworm infection among students of Mendera elementary school, Jimma, 2010

Predictors of Hookworm Infections	B	P-value	Adjusted OR	95.0% C.I.	
				Lower	Upper
Sex (Male)			1.00		
Sex	0.09	0.790	1.09	0.58	2.06
Age	0.15	0.066	1.16	0.99	1.35
Grades of the students	-0.29	0.002*	0.75	0.62	0.90
Finger nail status (trimmed)			1.00		
Finger nail status	-0.47	0.122	0.63	0.35	1.13
Latrine usage (Always)			1.00		
Latrine usage	-0.20	0.772	0.82	0.22	3.08
Hand washing after defecation (Yes)			1.00		
Hand washing after defecation	0.56	0.282	1.75	0.63	4.84
Eating uncooked vegetables (No)			1.00		
Eating uncooked vegetables	0.28	0.383	1.32	0.71	2.45
Water for drinking (Tap water)			1.00		
Other* source for drinking	-0.86	0.122	0.42	0.14	1.26
Shoe wearing habit (Always)			1.00		
Shoe wearing habit	-1.17	0.444	0.31	0.02	6.25

Other* like Birrie, spring, combination of Tap and Birrie, Tap and spring

## Discussion

This study indicated that *A. lumbricoides, T. trichiura,* Hookworms and *H. Nana* were intestinal helminths parasitizing school children of Jimma town with varying degree of magnitude. In fact endemicity of intestinal parasitosis has long been established by the several studies conducted in the study area with different prevalence reported such as Amare *et al.* 2007, Ali *et al.* 1999, and Haile *et al.* 1994 [17, 21,22]. Of the 715 examined students 346 were infected with one and more than one intestinal parasites. This is an indication for the rampant existence of helminthic infection among elementary school attending population of Jimma town in general and soil transmitted helminthic infection in particular. Despite the existence of high water and latrine coverage in the town, this finding seems to be higher [18, 19]. This could be attributed to poor sanitary facility of the school as well as poor personal hygiene practice of the students.

The overall prevalence of intestinal parasitism in this study was 48.4% (346/715), that is nearer to 50% of the examined school children. Depending on the above finding it is possible to say that if 100 school children had their stool examined, the likelihood of obtaining 50 children or less with at least one intestinal helminth is high. With our simple observation, students were consuming locally made biscuits such as in local language *''Mutebek'', ''Pastie'' and ''Bombolino''* as well as locally homemade ice cream in local language *''Jelaty''* by their unwashed hands during break time. Possibly this could be the exposing factor for the existing high prevalence of helminthiases in the area. The predominant parasite encountered in this prevalence study was *A. lumbricoides* which accounts 23.6% (169/715). This prevalence is almost in agreement to the prevalence of ascariasis found in Wonji-Shoa Sugar Estate 22.2% [23]. But it is lower when compared to the reported prevalence of Wondo Genet, Portoviejo city in Ecuador, Asendabo Elementary and Junior Secondary school, Delta State in Nigeria and South Gondar with prevalence rates of 83.4, 63.0, 56.4, 48.41 and 28.9% respectively. The most probable reason could be due to better personal hygiene practice of Mendera school children than Wondo Genet, Portoviejo in Ecuador, Asendabo, Delta State in Nigeria, and South Gondar School children [10, 11, 13, 16, 21]. In contrast, it is higher in comparison to previous studies: 6.4% among school children of Adwa, 18.5% among school-age pupils in rural areas of southern China. The possible reason could be that the environment of Jimma town may be more favorable for completion of the life cycle of the parasite such as fertile soil, humid and wet environment [12, 15].

*Trichuris trichiura* was the second most frequently encountered parasite in this study with a prevalence rate of 23.1% (165/715). This prevalence was higher than the prevalence obtained in South Gondar Zone 9.5%, Portoviejo in Ecuador 10%, Southern China 11.2%, Delta State in Nigeria 17.39% and Jiren Elementary and Junior Secondary School 18.6%. This is may be due to lack of adequate sanitary facility in Mendera Elementary and Junior Secondary School than the others [11, 13, 15, 16, 22,]. But it is very much lower in comparison to the reported prevalence from Wondo Genet 86.4%. Various reasons could be explained; such as the recently started de-worming after 2001, better hygienic status of Mendera school children than Wondo Genet school children and less favorability of Jimma soil type for the completion of the life cycle of *T. Trichiura* than Wondo Genet soil [10].

The third most prevalent parasite encountered in this prevalence study was Hookworm which was 9.4% (67/715). This was quite higher than the prevalence reported in Adwa 1% and Portoviejo in Ecuador 1.4% and also slightly higher than the prevalence reported in Babile town (6.7%). It might be due favorable environmental and soil type of Jimma town that favors the development of hookworm larvae than Adwa, Portoviejo in Ecuador and Babile town [12, 16, 24]. The 9.4% finding of the present study is lower than the prevalence obtained from studies in South Gondar Zone 12.9%, Southern China 14.7% and Delta State in Nigeria 29.76%. It could be explained by better socio-economic status of Jimma school children that might lead to the use of protected water source for drinking; because the water coverage of the town was 84%, and regular use of shoes. [11, 13, 15].

The least frequent helminthic parasite detected in the present study was *H. Nana* with a prevalence rate of 4.8% (34/715). In fact, the actual prevalence may be higher than this 4.8%. This is because Saturated Sodium chloride floatation technique by the McMaster method is best to detect ova *A. Lumbricoides* and ova of the two hookworms; but it is less sensitive to detect ova of other helminths including *H. Nana* [25]. Despite the fact that, this 4.8% is lower when compared to the prevalence of Hymenolepiasis found in Babile town (10.1%). This can be due to improved personal hygiene practice of Jimma school children than Babile school children, in addition to the reason mentioned above [24]. But it was higher in comparison to the prevalence reported in three localities of South Wello (Kembolcha, Bati and Mekaneselam) (1.3%). This could be explained by worse sanitary condition of Mendera Elementary School of Jimma town than the three localities in south Wello (Kembolcha, Bati and Mekaneselam) [26].

In our study, the predominant parasite encountered (*A. Lumbricoides*) and the second leading predominant parasite encountered (*T. Trichiura*) shares similar prevalence almost 23%. This could be explained by similarity exhibited by both nematodes in their mode of entry into the definitive host, human being, through ingestion of embryonated eggs from the environment. Furthermore, their eggs require similar environmentally conducive conditions for embryonation; these are warmth, moisture and shady environment in the soil. So, this could be another reason for their co-existence in the town of Jimma that ultimately results in similar prevalence's among the school children participated in this study.

Even though, the larvae of the two species of hookworms namely *Ancylostoma duodenale and Necator americanus* shares similar environmental conducive conditions for maturation in the soil like that of Ascaris and Trichuris, the prevalence obtained in this study (9.4%) is not congruent to that of Ascaris and Trichuris. This could be attributed to their mode of transmission, because hookworm infection is acquired through skin penetration by the filariform larvae in contrast to ingestion of embryonated egg in the case of Ascaris and Trichuris. In this study polyparasitism with STHs occurred in 70 students making the rate 9.8% of the total examined students and 21.5% of those who had STHs. This is in disagreement with a study done in Malaysia by Al-Mekhlafi MS *et al*. in 2006 with a reported prevalence rate of 72.6% for mixed infections [27]. The possible explanation could be the observed difference in sample size between ours and the study in Malaysia. This is because our sample size 715 was very much higher when compared to 281 of Al-Mekhlafi MS et al.

According to WHO report, triple infection was mainly caused by *A. lumbricoides, T. trichiura* and hookworm [28]. In the present study, the prevalence of single, double and triple infection by soil transmitted helminths were 35.8%, 9.1% and 0.7% respectively. Particularly the prevalence of triple infection caused by *A. lumbricoides, T. trichiura* and hookworm was 0.7% (5/715); and hence this is in accordance with WHO report [28].

With regard to infection intensity, from the ascariasis infected students, 92%, 6% and 2% were with light, moderate and heavy infection intensities respectively. This is in disagreement with a report from Adwa in which all Ascaris infected students were with light infections. It could be explained by the fact that students of Jimma might have frequent exposure to the parasite than students of Adwa that can contribute to harboring large worm burden of *A. lumbricoides* [12]. Intensity of infection is expressed as egg per gram of feces (EPG). So the arithmetic mean (±SD) egg count for *A. lumbricoides* was 1994.08 (±4305.488); Which is in contrast with the finding in Wondo Genet 7343 eggs per gram of stool [10].

Similarly, the arithmetic mean (± SD) egg count for *T. trichiura* was 429.09 (±658.900) egg per gram of stool; which is in agreement with the finding in Wondo Genet 461 eggs per gram of stool [10]. Of the trichuriasis infected students 92% and 8% were with light and moderate infection intensities respectively. Similarly, from the hookworm infected students 99% and 1% were with light and moderate infection intensities respectively. This indicated that, majority of the students were with light infection by soil transmitted helminths. But none of the students were infected by heavy infection of Trichuriasis and hookworms. It is also in accordance with the finding reported in rural Gambia by Palmer and Bundy who reported that, the infection is over-dispersed, with a minority of the population typically excreting large quantities of eggs while the majority has light infections, excreting very few eggs [14]. Generally of the total STH infected students nearly 1% (3/326) were heavily infected with STH. However, it is in disagreement with a report from Ecuador in the City of Portoviejo 8.5%. The fact that frequent exposure to STH is a key factor to develop heavy infection of soil transmitted helminthiases for those who remained untreated; hence, the probable reason could be that school children of Mendera Elementary school might have less exposure to STH than Ecuador school children in the City of Portoviejo. This showed that the absence of overdispersion of parasite number among children of school age in Jimma town. Despite the involvement of one school in the present study in relation the number of schools in the town; our study result (high prevalence and low intensity) as per the WHO guideline [20] enables us to forecast that the school population of Jimma is at medium risk for soil transmitted helminthiases. Because all schools in the town were characterized by nearly similar socio-economic status.

Intestinal nematodes have been identified as a major source of chronic ill-health, compromising the growth potential and intellectual achievements of children throughout the world. The mechanisms whereby cognitive impairment may occur have been suggested to be nutritional deficiency [29]. Iron deficiency has a strong association with impaired school performance and is common in children with high-intensity hookworm infections. Another possible mechanism is that the subclinical symptoms of heavy intestinal infection reduce attention at school [29]. Hence there should be an intervention among children of school age by means of regular de-worming to keep the infection intensity at low level [30].

Of all the predictors of Ascaris infection, sex and the habit of hand washing after defecation are significantly associated with ascariasis. Because logistic regression analysis showed that females were 2 times more likely to develop Ascaris infection than males as well as those students who do not wash their hands after defecation are 3 times more likely to develop Ascaris infection than those who wash their hands after defecation. The possible explanation for sex as a predictor is that high intensity Ascaris infection is associated with other intestinal nematodes [29]. Hence in our study most of the double infected students are females. Therefore this will explain why females are 2 times more likely to develop Ascaris infection than males. Similarly, of all the predictors of hookworm infection, grade of the study subjects is significantly associated with hookworm infection. Because logistic regression analysis showed that students of higher grade are 25% less likely to develop hookworm infection than students of lower grade. This is could be due to that higher grade students are more prone to protect themselves from hookworm infection, like by avoiding drinking unprotected water, avoiding walking on bare foot.

Gender, grade level, latrine usage pattern and the habit of washing hands after defecation were found to be the associated risk factors for soil transmitted helminths whereas age range was found to be the associated risk factor for Hymenolepiasis nana infection.

## Conclusion

In conclusion, nearly half of the school children examined were infected with one and/or more than one soil transmitted helminth(s). Those students who use latrine sometimes had significant Trichuriasis, than those who use always. Students who did not wash their hands after defecation were three times more likely to develop Ascaris infection than those who washed their hands after defecation. Therefore, measures like health information dissemination on the advantage of washing hands after defecation and on proper use of latrine should be taken into account to alleviate the problem.

### What is known about this topic

- Soil transmitted helminthic infections have been recognized as important public health problems in many developing countries;
- The greatest numbers of soil-transmitted helminths infections occur in tropical and subtropical regions of Asia, especially China, India and Southeast Asia, as well as sub-Saharan Africa;
- Although light infections are often asymptomatic, heavy infections cause an array of morbidities, including dietary deficiencies and delayed physical and cognitive development.

### What this study adds

- Nearly half of the school children were infected with at least one STHs and majority of the students had light infection of soil transmitted helminths;
- Female students were two times more likely to be infected by ascariasis than male students;
- All the heavy infections identified were due to ascariasis only.

## Competing interests

The authors declare that this is their original work and there is no competing interests.
